# Cardiac Remodeling in Fish: Strategies to Maintain Heart Function during Temperature Change

**DOI:** 10.1371/journal.pone.0024464

**Published:** 2011-09-07

**Authors:** Jordan M. Klaiman, Andrew J. Fenna, Holly A. Shiels, Joseph Macri, Todd E. Gillis

**Affiliations:** 1 Department of Integrative Biology, University of Guelph, Guelph, Ontario, Canada; 2 Faculty of Life Sciences, University of Manchester, Manchester, United Kingdom; 3 Department of Laboratory Medicine, Hamilton Regional Laboratory Medicine Program, Hamilton Health Sciences, Hamilton, Ontario, Canada; 4 Department of Pathology and Molecular Medicine, McMaster University, Hamilton, Ontario, Canada; Brigham & Women's Hospital - Harvard Medical School, United States of America

## Abstract

Rainbow trout remain active in waters that seasonally change between 4°C and 20°C. To explore how these fish are able to maintain cardiac function over this temperature range we characterized changes in cardiac morphology, contractile function, and the expression of contractile proteins in trout following acclimation to 4°C (cold), 12°C (control), and 17°C (warm). The relative ventricular mass (RVM) of the cold acclimated male fish was significantly greater than that of males in the control group. In addition, the compact myocardium of the cold acclimated male hearts was thinner compared to controls while the amount of spongy myocardium was found to have increased. Cold acclimation also caused an increase in connective tissue content, as well as muscle bundle area in the spongy myocardium of the male fish. Conversely, warm acclimation of male fish caused an increase in the thickness of the compact myocardium and a decrease in the amount of spongy myocardium. There was also a decrease in connective tissue content in both myocardial layers. In contrast, there was no change in the RVM or connective tissue content in the hearts of female trout with warm or cold acclimation. Cold acclimation also caused a 50% increase in the maximal rate of cardiac AM Mg^2+^-ATPase but did not influence the Ca^2+^ sensitivity of this enzyme. To identify a mechanism for this change we utilized two-dimensional difference gel electrophoresis to characterize changes in the cardiac contractile proteins. Cold acclimation caused subtle changes in the phosphorylation state of the slow skeletal isoform of troponin T found in the heart, as well as of myosin binding protein C. These results demonstrate that acclimation of trout to warm and cold temperatures has opposing effects on cardiac morphology and tissue composition and that this results in distinct warm and cold cardiac phenotypes.

## Introduction

The hypertrophic response, and the resulting change in cardiac function, differs if the increased load is due to a pathological condition or a physiological requirement [1], [2]. In general, pathological cardiac hypertrophy results in diastolic dysfunction that is associated with collagen fibrosis, although there is also a loss of cardiac contractility due, at least in part, to a decrease in actomyosin (AM) Mg^2+^ ATPase activity [3], [4]. In contrast, a physiological hypertrophic response results in improved contractile function associated with an increase in the Ca^2+^ sensitivity of the myocardium as well as an increase in the activity of AM Mg^2+^-ATPase [1], [3].There are also clear differences in the structure and function of hearts exhibiting pathological and physiological cardiac hypertrophy, which are the outcome of distinct molecular signaling pathways (reviewed in [1]).

One model system that has the potential to assist with our understanding of the functional basis of physiological hypertrophy is the response of the trout heart to thermal acclimation. Trout are ectothermic and remain active in waters that can seasonally vary between 4°C (winter) and 20°C (summer). Previous work has demonstrated that cold acclimation of a number of fish species, including trout, induces cardiac hypertrophy [5], [6], [7], [8], [9]. The increase in relative ventricular mass (RVM) is thought to help compensate for the influence of low temperature on contractile function by increasing the number of contractile units [9]. Furthermore, work by Graham and Farrell [9] has demonstrated that the absolute stroke work by the hearts of cold-acclimated trout was higher than that of hearts from warm acclimated trout. The hypertrophic response is thought to be triggered by the cold-induced increase in blood viscosity, which increases stress on the heart [9]. This change in morphology is associated with a down-regulation of collagen genes (protein was not investigated) [10] and an increase in contractility, with the rate of AM Mg^2+^-ATPase increasing [11]. However, the molecular basis of this latter change in function is unknown.

This study addressed two questions. First, is there an integrated response of the trout heart across multiple levels of organization to thermal acclimation? Second, does the response to warm and cold acclimation result in different cardiac phenotypes? To answer these questions we characterized changes in contractile function, tissue morphology and composition, as well as changes in the expression and phosphorylation of specific contractile proteins in fish that were acclimated to 4°C, 12°C and 17°C. Ca^2+^ activation of the cardiac tissue was measured by characterizing the activity and Ca^2+^ sensitivity of AM Mg^2+^-ATPase and 2D difference gel electrophoresis (2D-DIGE) was used to characterize changes in the cardiac proteome.

## Methods

### Thermal acclimation of rainbow trout

The rainbow trout (O*ncorhynchus mykiss*) used in this study were obtained from Rainbow Springs Trout Farm in Thamesford, Ontario. Prior to thermal acclimation, passive integrated transponder tags were implanted into the abdomen of the rainbow trout. Mixed sex animals (average mass  = 452 ± 16 g) were randomly divided into three 2000L environmentally controlled recirculation systems in the Hagen Aqua lab (University of Guelph) and then held at 12°C for two weeks. The acclimation protocol was as described by Vornanen et al. [10] where water temperature was changed by 1°C per day until it reached either 4°C or 17°C. One tank (control) was kept at 12°C. The trout were held at their acclimation temperatures for at least 8 weeks. Water was kept at normoxic levels with a 12 h light: 12 h dark photoperiod cycle. All fish were fed *ad libitum* for the duration of the experiment. All protocols were approved by the University of Guelph Animal Care Committee (protocol #06R010).

### Heart morphology - Embedding and sectioning of tissue

Trout were rapidly euthanized with a cranial blow and severing of the cerebral spinal cord. Whole hearts were removed, rinsed of blood with physiological saline (175.7 mM NaCl, 7 mM KCl, 1.9 CaCl, 1.1 mM MgCl, 10 mM Na^+^ pyruvate, 10 mM HEPES, pH 7.5 at 25°C) and then embedded in Shandon Cryomatrix (Thermo Scientific, Hampshire, UK) embedding medium. Serial 20 µm sections of ventricle (apex upwards) were collected on Superfrost slides, and stored at −80°C.

### Histology

Masson's trichrome staining protocol was applied to frozen trout ventricle tissue sections to stain muscle and connective tissue. Stains were prepared using the technique described in Vollmer [12] with all chemicals obtained from Sigma-Aldrich (St. Louis, MO, USA). Slides were placed in Bouin's solution overnight then dehydrated in 70% ethanol for 10 minutes repeated 3 times. Slides were then stained in Celestine Blue for 5 minutes, rinsed in distilled water (dH_2_0) and immersed in Cole's alum haematoxylin for 5 minutes. After 15 minutes washing in tap water, the slides were immersed in Acid Fuchsin for 10 minutes and rinsed in dH_2_0 until clear. Tissue sections were then placed in phosphomolybdic acid for 5 minutes, drained, and then placed directly into methyl blue for 5 minutes. Following rinsing in dH_2_0, slides were dehydrated in 70% ethanol for 2 minutes, then 90% ethanol for 2 minutes, then 100% ethanol for 2 minutes. Finally, tissue was prepared for mounting in clear solvent Histoclear twice for 5 minutes each. Slides were then mounted with glass cover slips in DPZ mountant (VWR International, Leicestershire, UK).

### Immunodetection of antibody staining

To delineate single myocytes from histological trabecular bundles, sequential sections were immunostained using an anti connexin 43 (Cx43) antibody. Cx43 is a cardiac gap junction protein which is localized to the cell membrane in cardiomyocytes [13].

A PAP pen (Sigma-Aldrich) was used to draw boxes on Superfrost Plus glass slides around pre-mounted frozen tissue sections. Slides were next fixed in 10% buffered formalin (Sigma-Aldrich) for 30 minutes before 3, 10 minute washes in 0.01 M PBS (phosphate buffered saline; Sigma-Aldrich), followed by 30 minute permeabilisation in 0.1% Triton-X100 (Sigma-Aldrich) in PBS. Slides were then washed 3 times in 0.01 M PBS for 10 minutes before blocking of non-specific sites using 1% BSA (bovine serum albumin; Sigma-Aldrich) in PBS for 60 minutes. Primary antibody (Cx43 (H – 150), Santa Cruz Biotechnology) in 1% BSA was then applied to individual squares (containing tissue section) and incubated at 4°C overnight. Primary negative controls involved applying 1% BSA without diluted antibody. The following day slides were washed 3 times for ten minutes in PBS before application of the secondary antibody (Donkey anti-mouse Cy3, Chemicon International, Harrow, UK) in 1% BSA and incubated for 60–120 minutes. Following a final series of 3, 10 minute PBS washes, slides were mounted with cover slips in Vectashield (H-1000; Vector Labs, Peterborough, UK), and sealed with nail varnish.

### Imaging and quantification

Images of stained tissue sections were taken using a Zeiss LSM 710 light microscope with a 10X objective lens for histology stained sections and the same microscope in a confocal setting was used to image Cx43 stained sections using a 63x lens. *Image J* was used to measure bundle perimeter, bundle area and compact layer thickness of Masson's trichrome stained sections. *ImageJ* “threshold colour” plugin in binary mode was used to quantify connective tissue density as well as extra-bundular space in Masson's trichrome stained sections. The connective tissue present in the spongy layer and compact layer was calculated as arbitrary units (A.U.). These values were determined for the two compartments in a consistent number of pixels taken strictly from images of either the spongy layer or compact layer. Within each compartment thresholding was used to assess the total pixels that were muscle and the total pixels that were connective tissue. The A.U. for connective tissue therefore represents the ratio of connective tissue present in the compartment in relation to muscle tissue.

### Animal sampling and myofibrillar isolation

Hearts were collected from fish acclimated at the same time as those used for the morphological studies. The ventricle was dissected, then rinsed in standard buffer (in mM, 60 KCl, 30 imidazole pH 7.0, 2.0 MgCl_2,_ 0.1 PMSF, 0.01 leupeptin HCl and 0.1 benzamidine [PMSF, leupetin and bezamidine will be referred to as the protease inhibitors]) to remove any blood and then weighed. Half the ventricle was freeze-clamped in liquid nitrogen (for AM Mg^2+^-ATPase measurements) and the other half was treated with 10% trichloroacetic acid (TCA) for 1 minute (for 2D-DIGE analysis) then freeze-clamped. All samples were then stored at −80°C. Myofibrillar proteins were isolated as by Churcott et al. [14]. A Lowry assay was then used to determine the protein concentration (Sigma-Aldrich).

### Actomyosin Mg^2+^ - ATPase activity

AM Mg^2+^-ATPase was measured at 7°C and 17°C as described by [14]. 7°C was used as the low assay temperature as results were not repeatable when the assay was completed at 4°C. It is thought that EGTA, used to buffer Ca^2+^, was coming out of solution at 4°C. Free Ca^2+^ concentrations were determined using Maxchealator version 8 [15] as in Gillis et al. [16], adjusting for assay temperature and pH. Purified myofilament proteins (100 µg at 17°C and 150 µg at 7°C) were incubated in the activating solutions for 3 minutes at 17°C and 5 minutes at 7°C. The reaction was quenched with 500 µl of 10% TCA and then centrifuged for 3 minutes at 14,200 g. Phosphate levels were measured as in [17]. The hearts used for the AM Mg^2+^-ATPase measurements and 2D DIGE studies were not identified as being from either male or female fish. The resulting data was therefore not sex specific. However, for all AM Mg^2+^-ATPase measurements the standard error of the means was less then 15% (Table 1). This indicates that the enzyme activity and Ca^2+^ sensitivity was relatively consistent between all tissue samples within each treatment.

**Table 1:** Effect of acclimation temperature and assay temperature on the function of actomyosin Mg^2+^-ATPase in rainbow trout ventricle.

Assay temperature	V_max_(nmol Pi/(min · mg protein))	Q_10_	pCa_50_	Hill coefficient
**Cold acclimated (4°C)**				
17°C (n = 6)	81.11±10.96 *^,#^	2.27	6.76±0.07 *	1.27±0.10 *
7°C (n = 6)	35.68±5.50 *^,‡^		6.40±0.07 *	1.78±0.29
Control (12°C)				
17°C (n = 8)	60.54±5.03 ^†^	2.43	6.74±0.11^†^	1.84±0.19 *
7°C (n = 7)	24.92±2.90 ^†^		6.41±0.11^†^	1.59±0.22
**Warm acclimated (17°C)**				
17°C (n = 9)	54.82±5.90^ §,^^#, ‡^	2.06	6.56±0.07	1.42±0.11
7°C (n = 8)	26.66±3.40 ^§^		6.24±0.15	1.21±0.12

Note: Data are mean ± SEM. Values with the same symbol in the same column are significantly different from each other (p<0.05).

### 2D Difference Gel Electrophoresis (2D-DIGE)

2D-DIGE was performed to detect cardiac myosin binding protein C (cMyBP-C), skeletal troponin T (sTnT), cardiac troponin T (cTnT), regulatory light chain (RLC), tropomyosin (Tm), myomesin, and cardiac troponin I (cTnI) as described by Yuan et al. [18]. It has been demonstrated that within a string of spots (identified on a 2D gel) for each of these proteins, that the spot(s) located at the basic end are in the non-phosphorylated state while those closer to the acidic end are phosphorylated [18], [19], [20]. We have previously demonstrated that cMyBP-C, RLC, TnT and TnI can be phosphorylated in trout cardiac muscle [21].

### 2D-DIGE - Protein Extraction

Frozen trout myocardial tissue was ground to a powder using a mortar and pestle super-cooled with liquid nitrogen and dry ice. Approximately 100 µg of tissue was weighted out into a pre-cooled 1.5 mL micro-centrifuge tube and then 10 volumes (volume/mass) of lysis buffer (7 M urea, 2 M thiourea, 4% CHAPS, 30 mmol/L TRIS, pH 8.5) were added. Samples were then sonicated on ice using a Vibra Cell sonicator (Sonics and Materials INC, Danbury USA) for 3X 10 seconds and incubated on a rocking platform at 4°C for one hour. This was repeated and the samples were sonicated a third time before centrifugation at 16,200 g for 30 min at 4°C. The supernatant was transferred to a new tube and then the protein concentration was quantified using a Bradford assay (BIO-RAD) with the standards made in lysis buffer. Protein concentrations were then adjusted to 5 mg/mL then snap frozen using liquid nitrogen and stored at −80°C until fluorescent labeling.

### Fluorescent CyDye labeling

Protein samples prepared above were thawed and 10 ul (50 ug) of protein was labeled with 200 pmol of either Cy3 or Cy5 flourescent dye according to manufacturers instructions (GE Healthcare, Uppsala, Sweden). A pooled standard was created by mixing equal parts of all samples to be included in the analysis and then labeled with Cy2. The reaction was carried out for 30 min on ice in the dark and then stopped with the addition of 1 µl of 10 mM L-lysine and a further 10 min incubation in the dark. Samples were stored in the −80°C until use.

### Isoelectric focusing (IEF)

IEF was performed on an IPGphor 3 unit with a cup-loading manifold (GE Healthcare). First 18 cm Immobiline DryStrips, pH 3–10 non-linear (NL) and 7-11NL (GE Healthcare) were rehydrated in an Immoboline DryStrip Reswelling tray (GE Healthcare) with 340 µl of DeStreak buffer (GE Healthcare) with the addition of 65 mM dithiothreiotol [DTT] and 1.5% IPG buffer for 10–12 hours. The DryStrips were then wiped of excess oil and placed in the IPGphor 3 unit. A total volume of 120 µl of sample (5 µl of Cy3, Cy5 and Cy2 labeled proteins) in DeStreak buffer containing 65 mM DTT and 1.5% IPG buffer was then loaded into a focusing cup. The entire IEF tray was then filled with mineral oil. The protocol for isoelectric focusing of the pH 3–10NL strips was 50 V for 20 min, 500 V for 2 h, 1000 V gradient for 1 h, 8000 V gradient for 3 h and 8000 V for 60000 V-h. For the pH 7–11NL strips the protocol used was 50 V for 20 min, 500 V for 3 h, 1000 V gradient for 1 h, 8000 V gradient for 3 h and 8000 V for 60000 V-h. The IPGphor 3 unit has a light excluding cover and temperature was maintained at 20°C during the focusing protocol. The maximum current achieved during the focusing protocol was 50 µA/strip. At the end of focusing all strips were stored at −80°C until the second dimension was performed.

### SDS-PAGE

Gradient gels, 8–16% polyacrylamide (8% (w/v) acrylamide, 375 mM Tris-Cl, pH 8.8, 0.1% (w/v) SDS, 0.0021% N,N,N',N'-tetramethylethylenediamine [TEMED] and 0.1% ammonium persulfate [APS]; 16% (w/v) acrylamide 375 mM Tris-Cl, pH 8.8, 0.1% (w/v) SDS , 0.00043% TEMED, 0.05% APS and 8.5% glycerol), were made using the Ettan DALT*six* gradient maker (GE Healthcare) and left to polymerize for 2 hours with water saturated 1-butanol laid overtop of each gel. A 5% acrylamide stacking gel was added to the top of each gel before use.

After IEF the strips were placed in SDS equilibration buffer (75 mM Tris, pH 8.8, 6 M urea, 30% (v/v) glycerol, 2% (w/v) SDS) for pH 7–11NL strips or with 5% SDS (w/v) for pH 3–10 strips, with 1% (w/v) DTT and gently rocked for 15 minutes. The strips were then rinsed and placed into SDS equilibration buffer containing 2.5% (w/v) iodoacetamide and gently rocked for 15 minutes. The strips were then rinsed with SDS running buffer and placed on top of a polyacrylamide gel cassette and secured with 0.5% agarose solution with trace amounts of bromophenol blue. 1X SDS running buffer was placed in the bottom chamber of the DALT*six* electrophoresis unit and 2X SDS running buffer was loaded into the top chamber. The protocol used for running the gels was – 1W for 30 minutes, 2W for 30 minutes and then 17W x the number of gels in the DALT*six* unit for 4–6 hours. The protocol was terminated when the dye front moved past the bottom of the gel. Gels were then imaged using a Typhoon 9400 Scanner (GE Healthcare). Cy2 images were scanned using a 488 nm laser and a 520 nm band pass (BP)-30 emission filter. Cy3 labeled proteins were visualized with a 532 nm laser and a 580 nm BP-40 emission filter and Cy5 labeled proteins were scanned with a 633 nm laser and 670 nm BP-30 emission filter. All gels were scanned at a resolution of 100 microns. 2D-DIGE gels were then analyzed using DeCyder software (GE Healthcare).

### Preparative gels

Gels for spot picking were prepared and run as described above but with non-labeled cardiac proteins. IEF was performed with 250–500 µg of cardiac proteins that were diluted with DeStreak to a total volume of 100 µl. After the second dimension SDS-PAGE gels were stained for total protein with SYPRO-ruby, as per manufacturer's instructions (Invitrogen). Gels were then imaged using the Typhoon (GE Healthcare) and then spot picking was performed using the Ettan spot picker (GE Healthcare) or by hand.

### Protein identification

The picked spots underwent in-gel digestion and the resultant peptides analyzed using a LC/MSD Trap Ultra 6330 Mass Spectrometer (Agilent) as previously described [22]. The obtained spectra were then analyzed using Spectrum Mill MS Proteomics Workbench (Agilent). The spectra were run against a reptile and fish subset of the National Center for Biomedical Information (NCBI) Protein Database. Proteins IDs with at least two peptides and with Distinct Summed MS/MS search scores of at least 25 were chosen as true hits.

### Statistical Analysis

Data are presented as mean ± SEM. Relative ventricular mass [%,  =  ((heart mass / body mass) * 100)], muscle bundle area, connective tissue density and compact layer thickness were analyzed using a two-way (main effects temperature and sex) analysis of variance (ANOVA) followed by Tukey post-hoc tests. Effects of thermal acclimation on AM Mg^2+^-ATPase and protein expression were analyzed with a one-way ANOVA with a Tukey post hoc test. Effects of assay temperature on AM Mg^2+^-ATPase within an acclimation group were analyzed with a Student's t-test (SPSS statistics 19). Data expressed as a percentage were first arcsine square root transformed before analysis.

## Results

### Changes in ventricle size

**Figure 1:**
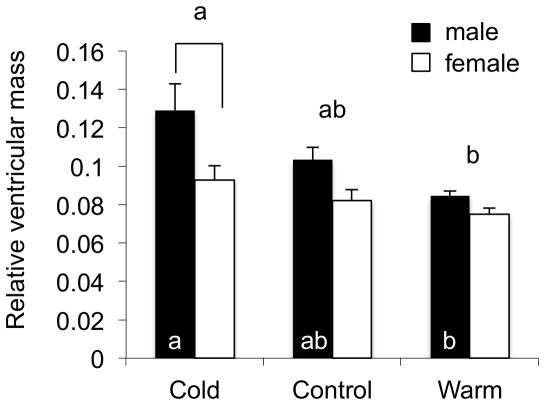
The effect of acclimation temperature on ventricle size. Cold (4°C) acclimated male rainbow trout had larger ventricle to body mass ratios compared to warm acclimated (17°C) male trout but not control (12°C) trout. Furthermore, cold acclimated male trout had larger ventricle to body mass ratios compared to female trout. Trout were acclimated for a minimum of 2 months, *N* = 15. Relative ventricular mass: ((heart mass / body mass) * 100). Values are mean ± SEM. Brackets, if present indicate significant differences between sexes at the same acclimation temperature. Different letters above the bars indicate significant difference between acclimation groups. Different letters within the bars for male fish indicate significant differences between acclimation temperatures (p<0.05).

There was no difference in the body mass of the cold, warm or control groups. These values were 713.2±47.9 g (cold), 749.5±24.2 g (warm), and 851.9±57.5 g (control). The average ventricle mass of the cold acclimated fish was 0.75±0.04 g. This value is not different from that of the control group (0.79±0.05 g) but it is significantly greater than that of the warm acclimated group (0.59±0.03 g). There was no difference between these values for the control and warm acclimated groups. When ventricle mass were standardized to whole animal mass the relative ventricular mass (RVM) of cold-acclimated males was 1.5 fold greater than that of the warm acclimated males. There was no change in RVM of the female fish with either cold or warm acclimation (Fig. 1B).

### Changes in the muscle bundle cross sectional area

**Figure 2:**
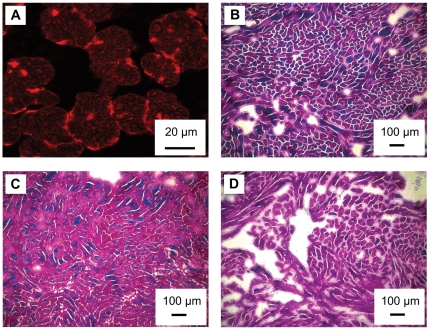
Masson's trichrome stained sections of ventricular spongy layer from thermally acclimated rainbow trout. **(**A**)** Cx43 staining at high magnification (63x). This image shows both the cross section of trabecular bundles (∼25 µm) and individual cardiomyocytes that make up the bundles (∼5 µm). (B) cold, 4°C, (C) control, 12°C, (D) warm, 17°C where pink/purple is muscle, blue is connective tissue and white or very pale pink is “extra bundular” space.

**Figure 3:**
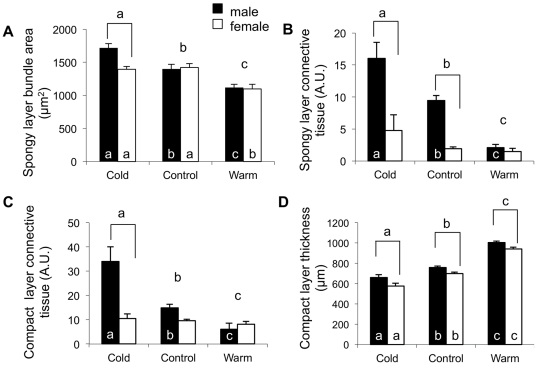
Quantification of Masson's trichrome stained sections of ventricular myocardium from thermally acclimated rainbow trout. Representative images are provided in Figs. 2 and 3. (A) Cold acclimation caused a significant increase in bundle area in the hearts of male fish. (B) Warm acclimation caused an increase in compact layer thickness in the hearts of both male and female fish. In addition, the thickness of the compact layer in the hearts of male trout was significantly greater than that of female fish in all three experimental groups. (C) The hearts of cold acclimated male trout had significantly more connective tissue in the spongy layer than that of either control or warm acclimated male fish. (D) Cold acclimation of male trout caused an increase in connective tissue content in the compact layer compared to controls while warm acclimation of the male trout caused a decrease in connective tissue content compared to controls. For panels C and D the amount of connective tissue present in the spongy layer and compact layer is presented as arbitrary units (A.U.) representing the ratio of connective tissue present in the compartment in relation to muscle. Values are mean ± SEM. Brackets, if present indicate a significant difference between sexes within an acclimation group. Different letters above the bars indicate a significant difference between acclimation groups. Different letters within the bars, if present, indicate significant differences between acclimation temperatures when each sex is analyzed separately (p<0.05).

The Cx43 immunoflorescence revealed that the Masson's trichrome was staining muscle bundles and not individual cardiomyocytes (Fig. 2A). As such, muscle bundles were used as a measure of the amount of cardiac muscle present in the two dimensional plane (Figs. 2B, 2C, 2D). The cross sectional area of muscle bundles in the spongy myocardium was 1.2 and 1.5 fold greater in the cold-acclimated fish compared to the control and warm-acclimated hearts, respectively (Fig. 3A). Warm acclimation caused a 0.8 fold decrease in the muscle bundle cross sectional area of both male and female fish (Fig. 3A). There was no difference between males and females in this measurement in the control and warm-acclimated groups (Fig. 3A).

### Connective tissue

**Figure 4:**
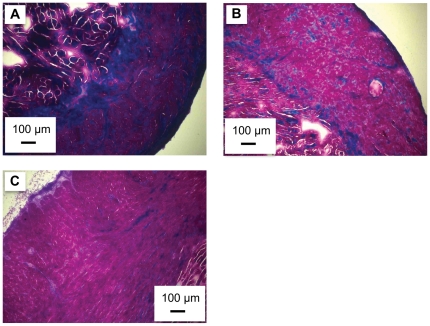
Masson's trichrome stained sections of ventricular compact layer from thermally acclimated rainbow trout. **(**A) cold, 4°C, (B) control, 12°C, (C) warm, 17°C where pink/ purple is muscle, blue is connective tissue and white or very pale pink is “extra bundular” space.

Cold acclimation caused the amount of connective tissue to increase in the hearts of the male trout. This increase, relative to control hearts, was 1.7 fold in the spongy layer (Fig. 3B), and 2.5 fold in the compact layer (Fig. 3C). Conversely, warm acclimation caused a decrease in the connective tissue of the male heart (Fig. 2 and Fig. 4). The connective tissue content in the spongy layer and compact layer of the warm acclimated male fish was 10 and 30%, respectively, of that measured in the control fish (Fig. 3B and Fig. 3C). As a result of the changes with thermal acclimation, connective tissue content was 12.4 and 8.1 fold greater in the spongy and compact layers, respectively, in the hearts of cold vs. warm acclimated male trout. In comparison, there was no influence of cold or warm acclimation on the connective tissue content in female hearts (Fig 3B and 3C). Finally, the connective tissue content in the cold acclimated male hearts was greater than that in the cold acclimated female hearts. This difference was 3.37 fold in the spongy layer (Fig 3B) and 3.23 fold in the compact layer (Fig 3C).

### Remodeling of the compact layer

The thickness of the compact layer in male and female fish increased with warm acclimation and decreased with cold acclimation, relative to control fish (Fig. 3D and 4). The changes caused by warm and cold acclimation results in the compact layer thickness of the warm acclimated fish being 1.6 fold that of the cold acclimated fish. The percentage change in thickness was similar for male and female fish. Additionally, the thickness of the compact layer of the male fish hearts was significantly greater than that of the female fish at each acclimation temperature (Fig. 3D).

### Actomyosin Mg^2+^-ATPase

**Figure 5:**
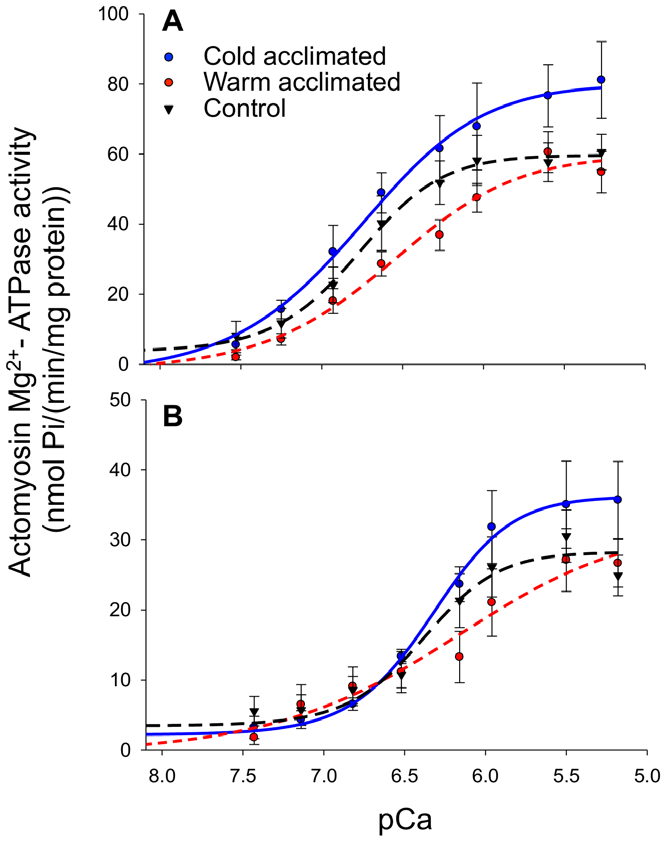
The effects of thermal acclimation on the actomyosin Mg^2+^-ATPase activity from trout ventricle. A) When measured at 17°C the maximal activity of cardiac AM Mg^2+^-ATPase was higher in cold acclimated trout than in warm acclimated trout. There was, however, no difference in Ca^2+^ sensitivity between acclimation groups at 17°C. (B) In contrast, when measured at 7°C there was no difference in the maximal activity of cardiac AM Mg^2+^-ATPase from all three experimental groups. Values are mean ± SEM for *N* = 6-9 at each temperature. A summary of this data is presented in Table 1.

We measured AM Mg^2+^-ATPase activity for all 3 acclimation groups at both cold (7°C) and warm (17°C) assay temperatures to assess the effect of thermal acclimation on contractile function. When measured at 17°C the maximal activity rates of this enzyme from the cold acclimated fish was 1.5 fold that from the warm acclimated group. However, there was no difference in the Ca^2+^ sensitivity of AM Mg^2+^-ATPase between the three acclimation groups (p<0.05, Fig. 5A and Table 1). Furthermore, at 17°C the Hill coefficient, used as a measure of cooperativity, was significantly less in cold than control fish (p<0.05). In contrast, when AM Mg^2+^-ATPase was measured at 7°C, there was no difference in the maximal rate or Ca^2+^ sensitivity or cooperativity.

All three groups had significantly lower maximal rates of AM Mg^2+^-ATPase activity at 7°C than at 17°C (p<0.05, Table 1; Fig. 5B and Table 1). In addition, the Ca^2+^ sensitivity of the myofilaments from the cold acclimated hearts and control hearts was significantly reduced at 7°C compared to at 17°C. There was, however, no significant difference in the Hill coefficients between the two assay temperatures in any of the acclimation groups (Table 1). Finally, comparison of the AM Mg^2+^- ATPase data from the warm acclimated trout assayed at 17°C with that from the cold acclimated trout assayed at 7°C suggests that some thermal compensation has occurred. Evidence for this is that while the maximal rate of enzyme activity from the cold acclimated trout were 0.65 fold that of the warm acclimated trout assayed at 17°C, there was no difference in the Ca^2+^ sensitivity of the AM Mg^2+^- ATPase (Table 1).

### 2D-DIGE

**Figure 6:**
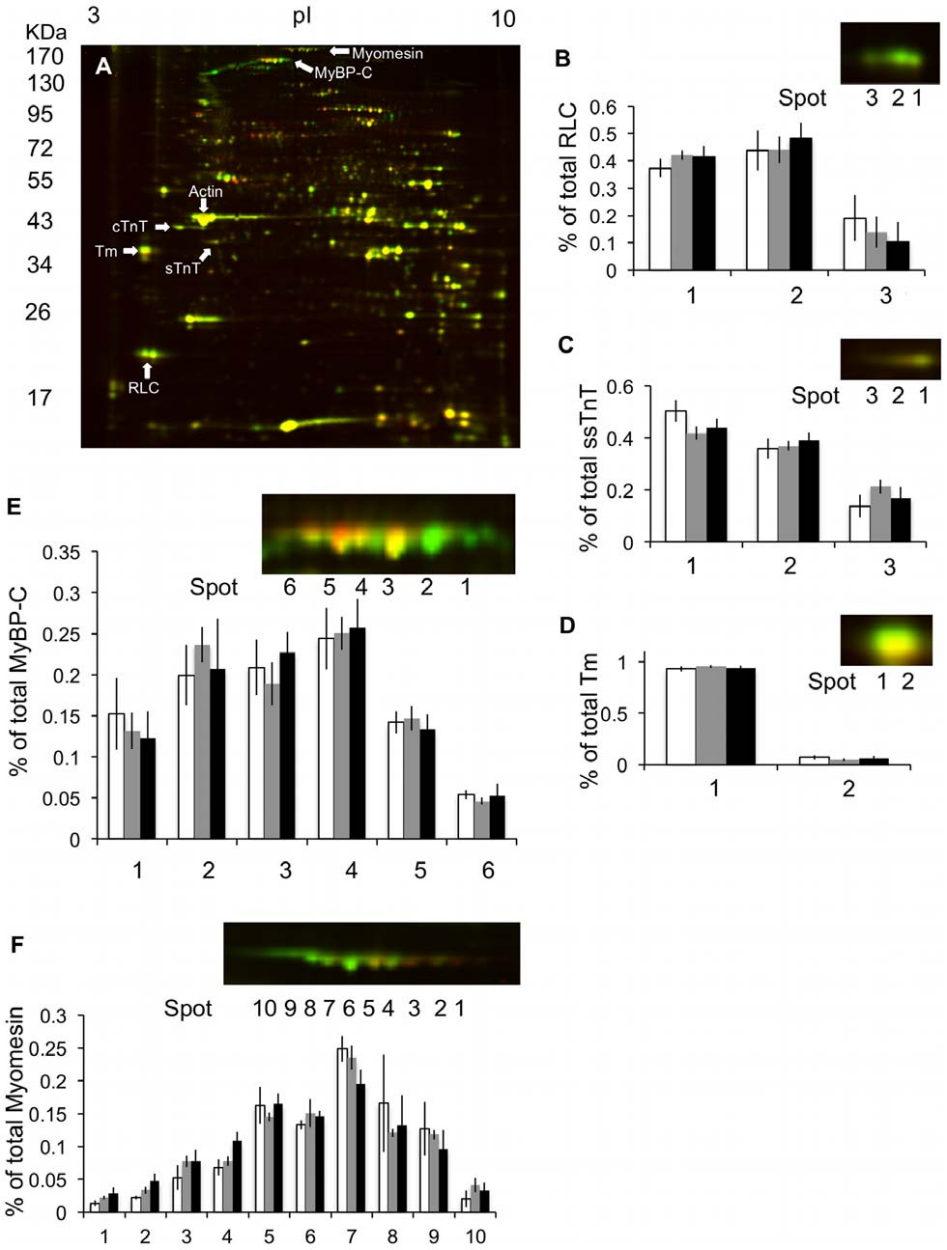
2D-DIGE analysis of cardiac contractile proteins from thermally acclimated trout. Thermal acclimation did not cause a change in the isoform expression of the identified contractile proteins in the trout ventricle. There was also no detected change in phosphorylation state of any of these proteins between experimental groups. (A) Representative 2D-DIGE analysis of cardiac proteomic changes with thermal acclimation. Proteins extracted from warm and cold acclimated trout were labeled with Cy-3 (green) and Cy-5 (red), respectively. The proteins were focused in the first dimension with an 18-cm, pH range 3–10NL, IPG strip. Superimposed Cy3/ Cy5 image is shown. Protein spots identified: cMyBP-C, myomesin, cardiac troponin T (cTnT), slow skeletal TnT (ssTnt), actin, tropomyosin (Tm) and regulatory light chains (RLC) are indicated. The phosphorylation state of (B) RLC, (C) ssTnT, (D) Tm, (E) cMyBP-C or (F) myomesin were not significantly affected by thermal acclimation. The most basic spot identified in a string of proteins is labeled spot 1. (White bars – cold acclimated, grey bars –control and black bars – warm acclimated).

A representative 2D-DIGE gel comparing Cy5-labeled cold-acclimated trout and Cy3-labeled warm-acclimated trout is shown in Fig. 6. Table 2 lists the protein ID data produced via the mass spec analysis. There were 6 spots identified on the 2D gels as cMyBP-C (Fig. 6A and 6E). While there was no significant change in the phosphorylation state of cMyBP-C it was observed that the spot density at the basic end of the cMyBP-C string for cold acclimated samples was greater than that of the warm acclimated samples (Fig. 6A and 6E). This suggests that cMyBP-C is less phosphorylated in cold than warm acclimated trout.

**Table 2:** Physical characteristics and analysis parameters of proteins identified in trout ventricle using mass spectroscopy analysis of protein spots picked from 2 dimensional protein gels.

Protein name	gi#	MW (kDa)	pI	Score	Coverage %
Cardiac myosin binding protein C	172072588	142	6.33	55.47	2
Myomesin	292616516	166	5.96	47.35	2
Actin, alpha	4902905	42	5.23	175.59	32
Troponin I	50540326	23	9.55	38.98	9
Troponin T, cardiac	23097290	34	5.19	30.07	6
Troponin T, skeletal	31377814	35	5.15	35.05	4
Myosin light Chain II	29725603	19	4.73	39.62	8
Tropomyosin	148231061	33	4.67	156.7	20

Note: gi#, accession number; MW, molar mass in kDa; pI, isoelectric point; Score, summed MS/MS search score; Coverage %, % of sequence covered by identified peptides.

**Figure 7:**
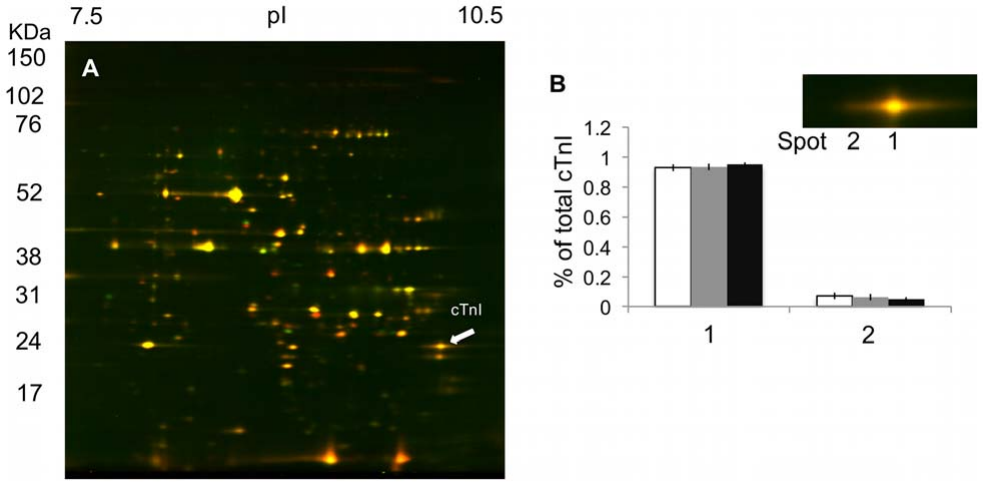
2D-DIGE analysis of the phosphorylation state of cTnI from thermally acclimated trout. (A) Representative 2D-DIGE analysis of cardiac troponin I (cTnI) in the cold and warm acclimated trout ventricle. Proteins extracted from cold and warm acclimated trout were labeled with Cy-5 (red) and Cy-3 (green), respectively and then focused in the first dimension with an 18-cm, pH range 7–11NL, IPG strip and 8-16% gradient SDS-PAGE for the second dimension. (B) No significant differences were observed in the phosphorylation state of cTnI (White bars – cold acclimated, grey bars –control and black bars – warm acclimated).

Two spots were identified as trout cTnI (Fig. 7), however thermal acclimation did not alter the relative proportion of these spots indicating that there was no change in the phosphorylation state of the protein (Fig. 7). Cold acclimation also did not alter the phosphorylation state of Tm or RLC (Fig. 6A, 6B and 6D). In the current study, two isoforms of TnT were differentiated using mass spectroscopy (cTnT and slow skeletal (ss) TnT) (Fig. 6A). Thermal acclimation had no affect on the ratios of these two isoforms with all three acclimation groups containing approximately 65% of cTnT and 35% ssTnT. Thermal acclimation also did not alter the phosphorylation state of cTnT. There may, however, be less phosphorylated ssTnT in the cold acclimated samples than the warm acclimated samples as there was less density at the most acidic spot (Fig. 6C).

## Discussion

This study demonstrates that the morphology and functional characteristics of the trout heart are actively regulated over the physiological temperature range of the animal. The changes in myocardium morphology, the amount of connective tissue within the heart and the activity level of AM Mg^2+^-ATPase demonstrate that the trout heart is remodeling across multiple levels of biological organization in response to acclimation to both low and high temperatures. The different effects of low and high temperature acclimation on cardiac morphology result in distinct cold and warm cardiac phenotypes in these animals.

### Changes in cardiac morphology with thermal acclimation

Cold and warm acclimation had opposite effects on heart morphology of the male fish. The increase in muscle bundle area with cold acclimation, compared to control samples indicates a hypertrophic response. These results are supported by previous studies on the influence of cold acclimation on heart size in carp [6], catfish [23], goldfish [24] and trout [8], [9], [25]. Previous work has also demonstrated that cold acclimation of trout causes an increase in cardiomyocyte perimeter and that this response is associated with an increase in muscle LIM protein (MLP) a molecular regulator, and marker, of cardiac myocyte hypertrophy [10]. The increase in muscle bundle area in the spongy layer of the heart of the male fish found in the current study, taken together with the results of these previous studies, indicates that cellular hypertrophy is the primary driver of the increase in RVM observed with cold acclimation. While cold acclimation has been previously demonstrated to cause cardiac hypertrophy in trout, this is the first known study demonstrating that warm acclimation causes a reduction in muscle bundle area in the spongy myocardium and in connective tissue content throughout the heart. These results suggest that the cardiac tissue is undergoing atrophy with warm acclimation.

The sex-specific effect of cold acclimation on heart morphology is likely due to differences in the levels of circulating sex hormones. Previous work on a variety of mammalian species [26], [27] as well as trout [28] demonstrates that testosterone is pro-hypertrophic. Conversely, estrogen has been demonstrated to be anti-hypertrophic [29], [30], [31]. The higher concentration of testosterone in male fish would therefore have enabled a greater hypertrophic response to cold acclimation. Conversely the higher concentrations of estrogen in female fish likely impeded a hypertrophic response.

### Changes in the levels of connective tissue with thermal acclimation

In mammals, differences in the amount and distribution of connective tissue are well documented between pathological and physiological hypertrophic hearts [1], [32], [33], [34]. Interestingly, we observed that the hypertrophic response of cold-acclimated trout hearts occurred in conjunction with an increase in connective tissue. This hypertrophic response is likely due to increased blood viscosity leading to an increased hemodynamic load on the ventricle. In rats an increase in hemodynamic load, initiated by aortic banding, causes an increase in collagen gene expression and the deposition of collagen fibroses in the left ventricle [35], [36], [37]. This is a chronic condition leading to cardiac dysfunction and eventual heart failure [1], [38], [39]. In the current study there was no sign of collagen fibrosis in the cold induced hypertrophic hearts. Previous studies in mammalian species have demonstrated that increases in connective tissue can stiffen the myocardium leading to diastolic dysfunction [1], [38]. However, how such a change influences the compliance of the trout myocardium is uncertain. The increase in connective tissue content found in the cold acclimated fish conflicts with previous work by Vornanen et al. [10], who showed a down regulation of the genes that encode for collagen and other extracellular matrix (ECM) proteins genes with cold acclimation. However, gene expression does not always coincide with protein expression [40].

Warm acclimation of the male trout caused a decrease in the amount of connective tissue throughout the ventricle. If this decrease was due to a proportional increase in the muscle content of the myocardium, then the RVM of the male fish should have increased. In addition there was no difference in body mass or growth rate (data not shown) between the warm and cold acclimated fish. This result suggests that connective tissue is being degraded in the heart of the male trout with warm acclimation. Having the ability to remove connective tissue from the myocardium makes the heart of male trout extremely plastic. Interestingly, this ability has not been documented in any other vertebrate. Further studies are required to dissect the regulatory pathways responsible for this unique ability. A logical place to begin looking is the role of microRNAs (miRNA), specifically the miRNAS-29 family regulating collagen deposition. Previous work has demonstrated that changes in the expression of these miRNA's influence the formation of the collagen matrix in mouse hearts and cultured fibroblasts [41].

### Changes in Spongy/Compact Layers

Similar to other salmonids, trout have a ventricle with an outer layer of tightly packed circumferentially arranged cardiomyocytes (the compact layer) which is adjoined via a transitional zone to the spongy layer made up of loosely connected trabecular sheets [42]. Blood can fill the inner-trabecular space (lacunae) increasing the ability of the ventricle to hold blood and therefore increase end-diastolic volumes [43]. An extreme example of this cardiac architecture is found in the Antarctic icefish (Channichthyidae) where a large spongy layer in conjunction with high ventricular compliance is thought to allow the heart to displace large blood volumes at a low rate and relatively low pressure [43], [44]. In the current study the decrease in the thickness of compact myocardium, combined with the increase in RVM suggests an increase in the mass of the spongy myocardium. As the amount of connective tissue in the compact myocardium of the male fish increased with cold acclimation by 2.5 fold, this infers that the loss of tissue thickness was due to a decrease in muscle content. Graham and Farrell [9] proposed that the increase in RVM caused by cold acclimation of trout enables force production by a greater number of contractile units in the spongy myocardium per heart beat and increases the volume of blood moved per contraction. This would help maintain force generating capacity and volume output at low temperatures, as well as compensate for the loss of muscle in the compact layer. This hypothesis is supported by the work of these authors demonstrating that stroke volume of cold acclimated trout (5°C) was equal or greater than that of warm acclimated trout (15°C) when measured at their respective acclimation temperatures [9].

The results of the current study demonstrating an increase in compact layer thickness with warm acclimation is supported by previous work by Farrell et al. [5] where it was found that the compact layer was thicker in fish acclimated to 15°C than in fish acclimated to 5°C. These authors correlated the increase in compact layer thickness with an increase in activity level at higher temperatures [5]. One hypothesis for the increase in compact layer thickness with warm acclimation is that it helps to offset the negative effects of warm temperature at the lower range of contraction frequencies. Previous work with trout by Farrell et al. [45] demonstrates that as experimental temperature is increased from 10°C to 15°C maximum power output and stroke volume decrease. This loss of function is thought to result in a loss of pressure-generating ability at temperatures above 15°C [45]. The increase in the thickness of the compact layer with warm acclimation may therefore be an attempt to compensate for these functional losses.

### Actomyosin Mg^2+^-ATPase as a Measure of Contractility

The higher rates of AM Mg^2+^-ATPase activity throughout the physiological Ca^2+^ range of the heart in cold acclimated trout would translate into higher rates of cross-bridge cycling. This finding is supported by previous work by Yang et al. [11], which showed that cold acclimated trout had higher rates of AM Mg^2+^-ATPase activity when assayed at 10°C. In the current study the maximum rate of AM Mg^2+^-ATPase activity was lower in the cold acclimated animals measured at 7°C than in the warm acclimated animals measured at 17°C. This indicates that there would be differences in the rate of cross-bridge cycling in the hearts from the two acclimation groups at their respective physiological temperatures. As the rate of cross-bridge cycling within a muscle translates into the rate of force generation [46], these differences in AM Mg^2+^-ATPase activity likely reflect differences in the ability of the hearts to contract at the different temperatures. However, there was no difference in the Ca^2+^ sensitivity of this reaction between the cold and warm acclimated groups. This means that the same amount of Ca^2+^ is required to initiate the contractile reaction and suggests that there is only partial compensation in the cold acclimated heart for the effects of low temperature on contractile function.

The number of protein spots identified as cMyBP-C on the trout heart 2D-DIGE maps is approximately half that reported for cMyBP-C on mammalian heart 2D-DIGE maps [18]. This is consistent with a recent study that suggests that teleost cMyBP-C contains half the number of phosphorylation sites as cMyBP-C from mammalian species due to difference in protein sequence [47]. In addition, the number of cTnI spots on the trout heart 2D-DIGE maps is also less than on maps from mammalian hearts [18], [19], [20]. This is because trout cTnI lacks the N-terminal extension found in mammalian isoforms that contain 2 phosphorylation sites [47].

Within the mammalian heart, the phosphorylation of cTnT [48] and cMyBP-C [49], [50], [51] has been shown to alter the rate of cross-bridge cycling and AM Mg^2+^-ATPase activity. In the current study, cold acclimation caused a slight, but not significant, decrease in the phosphorylation state of ssTnT and cMyBP-C. These results suggest that only small changes in the level of phosphorylation are required to cause a significant change in the functional characteristics of the trout contractile element. Alternatively it may be that the observed changes in AM Mg^2+^-ATPase activity are not due to a change in protein phosphorylation state, but to the expression of a novel protein isoform. Possibilities include TnI or RLC as each of these proteins are known to affect the rates of AM Mg^2+^-ATPase. The analysis of the DIGE maps did not find such an isoform but it is possible that we did not identify all isoforms of these proteins on the gels.

### Conclusions and Perspectives

Together our results demonstrate that the trout heart undergoes an integrative remodeling response as the animal acclimates to warm and cold temperatures. This response includes changes in cardiac morphology, tissue composition and function. These results also demonstrate that the morphological changes that occur with acclimation to cold and warm temperatures result in two distinct cardiac phenotypes. The cold induced cardiac hypertrophy is associated with an increase in muscle content and connective tissue, while the relative atrophy with warm acclimation is due to a reduction in muscle content and connective tissue. The increase in spongy layer with cold acclimation may represent a compensation for the reduction in the activity of AM Mg^2+^-ATPase. This morphological change increases the amount of contractile tissue present in the heart. Any reduction in contractile ability caused by the lower activity of AM Mg^2+^-ATPase may therefore not have as significant an effect on cardiac output. The opposing effects of warm and cold acclimation on connective tissue content is particularly interesting as it suggests that the increase in connective tissue associated with cold induced hypertrophy is reversible. Such ability has significant application to the study of cardiac disease, where cardiac hypertrophy leads to the permanent deposition of connective tissue, impaired function and eventual heart failure. Further investigations into the regulation of the remodeling response in the trout heart during thermal acclimation may, therefore, yield important insight into the pathology of cardiac hypertrophy.
